# Supplementary material for: Predicting visual function by interpreting a neuronal wiring diagram
Source: Nature. 2024 Oct 2;634(8032):113–23. doi: 10.1038/s41586-024-07953-5 (PMC11446822; doi:10.1038/s41586-024-07953-5)
Supplement: Supplementary file 6 — CRF and ERF predictions for individual TmY4 and TmY9 cells. Analogous to Supplementary Data 3, but for TmY target types. Shown are the top four monosynaptic pathways, the strongest pathway passing through each of the top ten intermediary types (ranking from Extended Data Fig. 7), and the trisynaptic pathway Tm1–TmY–Dm3–TmY (see the section entitled Prediction of spatial normalization). [file 41586_2024_7953_MOESM6_ESM.zip › DataS4/TmY9q/720575940654677665.pdf]

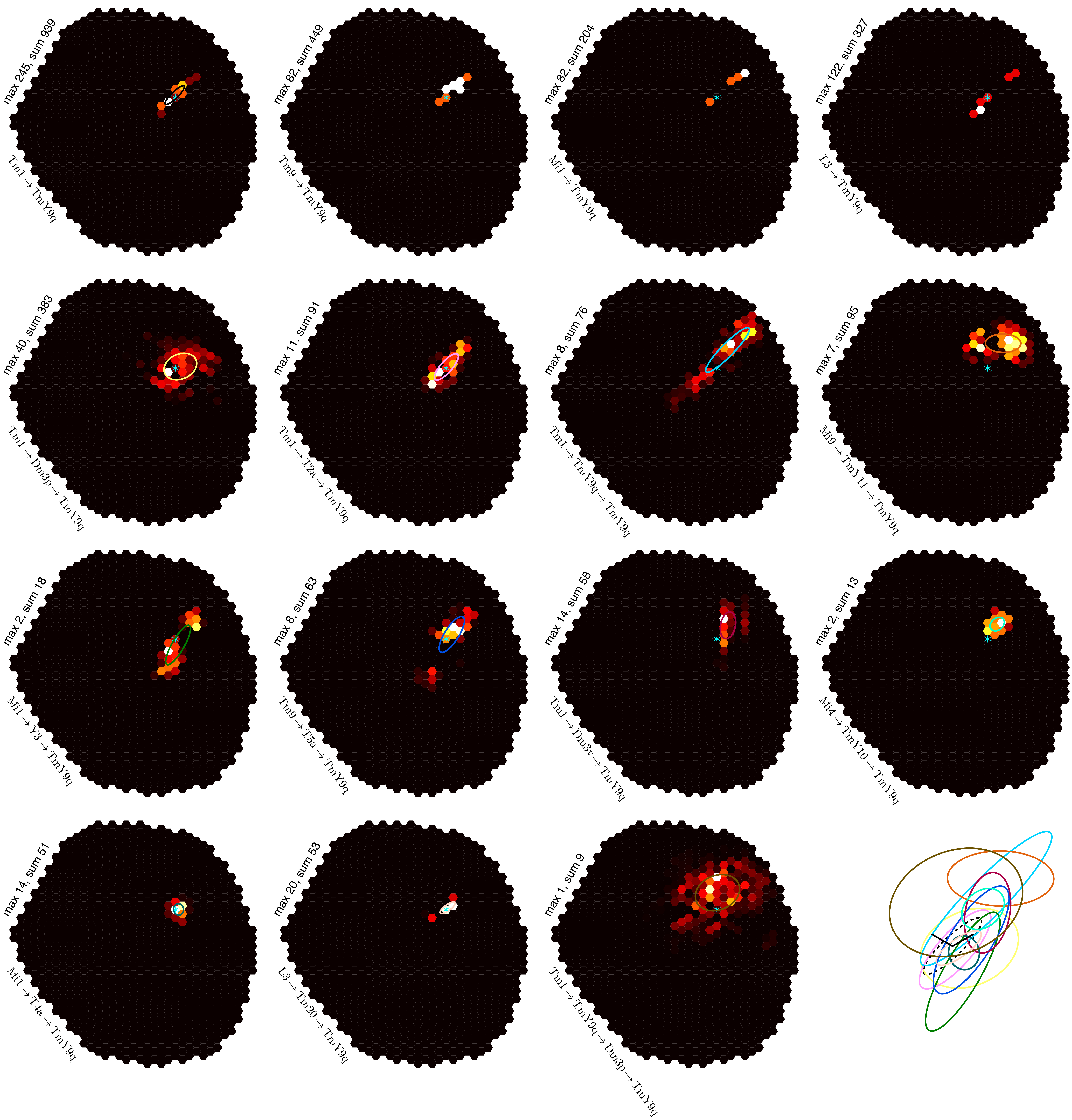
max 245, sum 939
Tm1 → TmY9q
max 82, sum 449
Tm9 → TmY9q
max 82, sum 204
Mi1 → TmY9q
max 122, sum 327
L3 → TmY9q
max 40, sum 383
Tm1 → Dm3p → TmY9q
max 11, sum 91
Tm1 → T2a → TmY9q
max 8, sum 76
Tm1 → TmY9q → TmY9q
max 7, sum 95
Mi9 → TmY11 → TmY9q
max 2, sum 18
Mi1 → Y3 → TmY9q
max 8, sum 63
Tm9 → T5a → TmY9q
max 14, sum 58
Tm1 → Dm3v → TmY9q
max 2, sum 13
Mi4 → TmY10 → TmY9q
max 14, sum 51
Mi1 → T4a → TmY9q
max 20, sum 53
L3 → Tm20 → TmY9q
max 1, sum 9
Tm1 → TmY9q → Dm3p → TmY9q
